# Advances in CAR T-cell therapy for treating patients with mantle cell lymphoma: a critical appraisal

**DOI:** 10.1097/JS9.0000000000000691

**Published:** 2023-09-02

**Authors:** Ruhul Amin, Ronald Darwin, Sandip Chakraborty, Abhijit Dey, Kuldeep Dhama, Talha Bin Emran

**Affiliations:** a Faculty of Pharmaceutical Science, Assam down town University, Panikhaiti, Gandhinagar, Guwahati, Assam; b School of Pharmaceutical Sciences, Vels Institute of Science Technology and Advanced Studies, Chennai; c Department of Veterinary Microbiology, College of Veterinary Sciences and Animal Husbandry, R.K. Nagar, West Tripura, Tripura; d Department of Life Sciences, Presidency University, Kolkata, West Bengal; e Division of Pathology, ICAR-Indian Veterinary Research Institute, Bareilly, Izatnagar, Uttar Pradesh, India; f Department of Pharmacy, BGC Trust University Bangladesh, Chittagong; g Department of Pharmacy, Faculty of Allied Health Sciences, Daffodil International University, Dhaka, Bangladesh

CAR T-cell (chimeric antigen receptor T-cell) has shown encouraging outcomes in the treatment of several cancers, including mantle cell lymphoma (MCL) that is a rare type of non-Hodgkin lymphoma (NHL) which begins in the lymph nodes. It is distinguished by cyclin D1 protein overexpression caused by a chromosomal translocation between the immunoglobulin heavy chain locus (IGH) and Cyclin D1 (CCND1) gene. In the case of MCL, excessive CCND1 is produced by B cells that are abnormal. Notably, CCND1 helps in the growth of B cells. Clinical characterization of MCL is done by its heterogeneity nature. The course of the disease ranges from indolent cases for which therapy is not essential for years to MCL that is highly aggressive in nature (requiring treatment), with the prognosis very limited. New targeted immunotherapy-based approaches, viz., CAR T-cell therapy, have already facilitated the improvement of the therapeutic options, particularly for the refractory or relapsing disease^1^.

CAR T-cell treatment includes genetically altering a patient’s own T cells to express a tumor-associated antigen-specific chimeric antigen receptor. Then, the CAR T cells are injected back into the patient, where they distinguish and kill cancer cells expressing specific antigens (Fig. 1). Notably, advanced immunotherapy with the use of CAR T cells is found to be fruitful for patients who suffer from MCL. Importantly, in comparison to other drugs that exist, CAR T-cell therapy is found to be successful in managing MCL resistance^2^. A feasible approach in the case of MCL that is re-occurring in nature is to target B cell antigens on the surface of the cells. This approach has provided scientists with significant responses in other cancers of B cells too.

**Figure 1:**
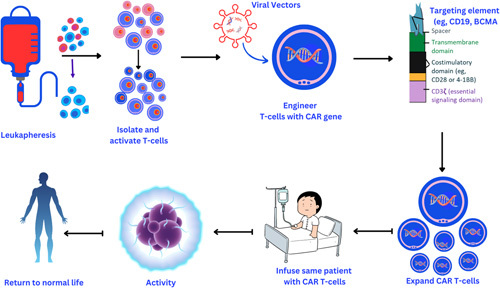
The phases of CAR T-cell (chimeric antigen receptor T-cell) therapy production, manufacture, and administration.

In the ZUMA-2 study, patients suffering from relapsed or refractory MCL when given axicabtagene ciloleucel (axi-cel) (an example of autologous cell therapy), a CD19-targeted CAR T-cell treatment, had a high overall response rate (ORR) of 91% and a complete response (CR) rate of 68%. These results prompted the USFDA to approve axi-cel for treating relapsed or refractory MCL^3^. The USFDA has also given approval to brexucabtagene autoleucel (brexu-cel/KTE-X19), a novel therapeutic approach involving CAR T cells. This therapy is particularly recommended for patients with MCL who have remained unresponsive to therapies previously or in the case of patients where the condition has relapsed. Approval has been given to this treatment on the basis of ZUMA-2 clinical trial outcome^2^. Brexu-cel/KTE-X19 elicited long-term responses with manageable safety in relapsed/refractory MCL patients, and may help individuals with high-risk features^4^. The safety and efficiency of brexu-cel therapy are quite noteworthy. It has got a remarkable rate of response in aged patients, patients having blastoid variant and greater Ki-67 index, and in those people with *TP53* mutation^1^.

The TRANSCEND NHL 001 study looked for the safety and effectiveness of another CD19-directed CAR T-cell treatment, lisocabtagene maraleucel (liso-cel), in patients having relapsed or refractory MCL. Treated patients revealed an ORR of 84% and a CR rate of 59%. Because of these findings, the FDA granted liso-cel expedited approval for treating relapsed or refractory MCL^5^.

Notably, USFDA has approved six CAR T-cell-based therapies for treating various blood cancers since 2017. The authorized therapies include ciltacabtagene autoleucel, idecabtagene vicleucel, and tisagenlecleucel, apart from axicabtagene ciloleucel, brexucabtagene autoleucel, and lisocabtagene maraleucel. Brexucabtagene autoleucel targeting the CD19 antigen is found to be most effective against cancer like MCL^6^. The other name of brexucabtagene autoleucel is Tecartus, which has got approval for use in patients with MCL who do not respond to other therapies or in which case there is a chance of recurrence. In certain patients, the drug may cause side effects that are serious and can potentially threaten life. Such side effects may include cytokine release syndrome, which can result in a high rise in temperature and symptoms that are similar to flu. The drug may also cause neurologic effects, because of which the patients may become comatose. Importantly, axicabtagene ciloleucel as well as tisagenlecleucel are the two CAR T-cell products that are CD19 targeted, and can be used for treating relapsed or refractory B cell lymphoma (large) that are aggressive in nature^7,8^. Efforts are being made to look into the possibility of combining CAR T-cell therapy with other therapeutic modalities to enhance MCL patient outcomes. Acalabrutinib is a BTK inhibitor that has been demonstrated to be effective in MCL^9^. Preclinical research has shown that combining acalabrutinib with CAR T-cell treatment has synergistic benefits in targeting MCL cells. Checkpoint inhibitors such as pembrolizumab and nivolumab have shown efficacy in relapsed or refractory MCL. Combining CAR T-cell treatment with checkpoint inhibitors may improve antitumor immune response. This combo technique is now being tested in clinical studies^10^. Synergism of CAR T cells with ibrutinib has been preliminary evidenced by a preclinical study involving xenograft models (mouse origin) of MCL. When ibrutinib is added to CTL019, there is augmentation of the preexisting potency of the function of the antitumor CAR T cell^6,11^.

CAR T-cell treatment is found to be linked to a variety of side effects, such as cytokine release syndrome (CRS), brain toxicity, and immune effector cell-associated neurotoxicity syndrome (ICANS). For overcoming such barriers, prompt identification and control of such side effects is critical for CAR T-cell therapy to be effective. Guidelines and algorithms have been created to help healthcare practitioners manage the toxicities associated with CAR T-cell treatment^12^.

The haplo-CAR T where derivation of the T cells is done from matched donors who are healthy and have blood relations (like children or siblings), has been found to be efficacious for patients with MCL that is refractory in nature. Proliferation of haplo-CAR T cells can take place *in vivo* effectively and has activity against cancer that is clinically significant. Moreover, any serious adverse effects are not observed^2^. In this context, it requires special mention of a case of MCL (refractory) in a patient wherein relapsing has been observed following chemotherapy in a conventional manner and autologous CAR T-cell therapy. Haplo-identical CAR T cells were received by the patient from her daughter without transplantation of allo-hematopoietic stem cells previously. Remission has been achieved by the patient partially, with the residual disease being minimal. Such case history is suggestive of the fact that haplo-CAR T-cell therapy can be found to be efficacious to control lymphoma, for which autologous CAR T-cell therapy is found to be a failure^13^.

In conclusion, CAR T-cell therapy is emerging as a viable therapeutic choice for individuals with relapsed or refractory MCL. A high response rate is provided by brexu-cel in refractory as well as relapsed MCL with a tolerable profile of safety. Stratifying patients as per their risk level, viz., *TP53* mutations and raised Ki-67 index, may prove to be useful in individualization of the approach of patients with MCL. Clinical studies have shown CAR T-cell treatment to have a high response rate, leading to its approval for therapeutic purposes. Ongoing research seeks to enhance the management of therapy-related toxicities and optimize treatment methods, including combination approaches. The efficacious nature of the CAR T cells, even in subgroups that are at high risk, may direct researchers to utilize it earlier in the course of management of MCL.

## Ethical approval

Not applicable.

## Sources of funding

No funding was received.

## Author contribution

R.A.: conceptualization, data curation, writing – original draft preparation, reviewing, and editing; R.D., S.C., and A.D.: data curation, writing – original draft preparation, reviewing, and editing; K.D.: writing – reviewing and editing and visualization; T.B.E.: writing – reviewing and editing, visualization, and supervision.

## Conflicts of interest disclosure

The authors declare that they have no conflicts of interest.

## Research registration unique identifying number (UIN)

1. Name of the registry: not applicable.
2. Unique identifying number or registration ID: not applicable.
3. Hyperlink to your specific registration (must be publicly accessible and will be checked): not applicable.

## Guarantor

Talha Bin Emran, PhD, Associate Professor, Department of Pharmacy, BGC Trust University Bangladesh, Chittagong 4381, Bangladesh. Tel.: +88 030 3356193, fax: +88 031 2550224, Cell: +88 01819942214; https://orcid.org/0000-0003-3188-2272.

## Data availability statement

The data in this commentary article is not sensitive in nature and is accessible in the public domain. The data is therefore available and not of a confidential nature.
